# Phenotypic selection in weedy radish and its bidirectional crop–weed hybrids across two contrasting environments

**DOI:** 10.1093/aobpla/plag010

**Published:** 2026-02-13

**Authors:** Román B Vercellino, Fernando Hernández, Sofia Tillería, Denise Simian, Ignacio Fanna, Alejandro Presotto

**Affiliations:** Departamento de Agronomía, Universidad Nacional del Sur (UNS)—Centro de Recursos Naturales Renovables de la Zona Semiárida (CERZOS)-CONICET, San Andrés 800, Bahía Blanca 8000, Buenos Aires, Argentina; Department of Botany and Biodiversity Research Centre, University of British Columbia, 2212 Main Mall, Vancouver, British Columbia, BC V6T 1Z4, Canada; Departamento de Agronomía, Universidad Nacional del Sur (UNS)—Centro de Recursos Naturales Renovables de la Zona Semiárida (CERZOS)-CONICET, San Andrés 800, Bahía Blanca 8000, Buenos Aires, Argentina; Departamento de Agronomía, Universidad Nacional del Sur (UNS)—Centro de Recursos Naturales Renovables de la Zona Semiárida (CERZOS)-CONICET, San Andrés 800, Bahía Blanca 8000, Buenos Aires, Argentina; Departamento de Agronomía, Universidad Nacional del Sur (UNS)—Centro de Recursos Naturales Renovables de la Zona Semiárida (CERZOS)-CONICET, San Andrés 800, Bahía Blanca 8000, Buenos Aires, Argentina; Departamento de Agronomía, Universidad Nacional del Sur (UNS)—Centro de Recursos Naturales Renovables de la Zona Semiárida (CERZOS)-CONICET, San Andrés 800, Bahía Blanca 8000, Buenos Aires, Argentina

**Keywords:** admixture, hybrid zone, crop-wild hybrids, selection differentials, selection gradients, linear selection, quadratic selection, functional traits, phenotypical traits, *Raphanus sativus*

## Abstract

Hybridization is a key mechanism in the adaptive evolution of weeds and invasive species. The evolutionary success of hybrids may be shaped by selection on functional traits, interacting with maternal genotype and ecological context. We performed experimental hybridization and common garden field experiments to assess phenotypic variation on functional traits, and the strength and direction of linear and quadratic selection acting on them, in bidirectional crop–weed hybrids and their parents of *Raphanus sativus*, across two contrasting environments: agrestal (agricultural) and ruderal (human-disturbed uncultivated area), over two years. Bidirectional hybrids exhibited overall greater values for size-related traits than their parents, with similar flowering time and no significant effects of cross direction. Selection acted on multiple functional traits through both linear and quadratic components, with broadly similar patterns across environments and cross types, although selection tended to be slightly stronger in hybrids than in weeds. Intraspecific crop–weed hybridization, regardless of cross direction, can increase weediness in weedy radish by enhancing plant size, thereby increasing competitive ability and potential interference with crops. Our findings highlight how hybridization and selection shape plant evolution, influencing the potential introgression of crop alleles into wild or weedy gene pools. Understanding the hybridization process and the mechanisms that control it is crucial for managing the evolutionary outcomes of crop-weed hybridization.

## Teaser

Crop–weed hybridization can rapidly alter weed evolution. Using weedy radish (Raphanus sativus), we show that first-generation crop–weed hybrids consistently develop larger plants than their parents in both agricultural and ruderal environments. Natural selection acted on multiple functional traits, mainly favouring increased plant size. These results indicate that intraspecific hybridization can enhance weediness by increasing competitive ability and facilitating crop allele introgression.

## Introduction

Plant hybridization is recognized as a key evolutionary process that can impact the evolution of weeds (Ellstrand et al. 2013, Vercellino et al. 2023a ). After hybridization occurs, offspring inherit alleles from both parent lines, and the permanent introgression will depend on the relative fitness of the hybrids compared to their progenitors (Westbrook and DiTommaso 2023, Hernandez et al. 2023). Hybrids often show intermediate and maladapted phenotypes, leading to reduced fitness relative to their parents (Mercer et al. 2006, Sahoo et al. 2010, Martin et al. 2019, Yue et al. 2021), with extreme cases resulting in nonviable or sterile offspring (Rieseberg et al. 2007). However, in other cases, hybridization can facilitate adaptation through increased genetic variation, evolutionary novelty, heterosis (hybrid vigour) and the dumping of genetic load, enhancing weediness and invasiveness (Schierenbeck and Ellstrand 2009, Hovick and Whitney 2014). Gene flow between crops and wild or weedy relatives can serve as a model to study hybridization between taxa with divergent life histories and its potential to drive rapid evolution in fitness-related traits (Ellstrand et al. 2013, Westbrook and DiTommaso 2023). There are multiple reports where spontaneous hybridization between crops and their wild/weedy relatives has driven increased weediness and invasiveness (Snow et al. 2010, Vigueira et al. 2019, Le Corre et al. 2020, Hernández et al. 2022, 2024). However, in other cases, hybridization appears not to affect weediness or invasiveness (Magnussen and Hauser 2007, Whitney et al. 2009, Martin et al. 2019, Page et al. 2019).

Hybrid performance relative to its parents is environment-dependent (Mercer et al. 2006, Campbell and Snow 2007, Hovick and Whitney 2014). Thus, the evolutionary outcomes of hybridization may differ between agrestal (agricultural) and ruderal (human-modified uncultivated) environments (Mercer et al. 2006, Mitchell et al. 2021). In agricultural habitats, wild or weedy species may benefit from acquiring crop traits, especially those related to resistance to herbicides, pests, or diseases, and tolerance to abiotic stresses (Merotto et al. 2016, Pandolfo et al. 2018a, Nam et al. 2020, Kreiner et al. 2022), but also from other typical crops traits like early flowering, rapid growth, and higher seed production (Campbell et al. 2009a, Presotto et al. 2017, Le Corre et al. 2020). However, the transfer of crop traits, shaped by human selection in agricultural environments, may result in maladapted crop–wild/weed hybrids in less selective habitats, such as ruderal environments, limiting their potential for adaptive evolution (Mercer et al. 2007, Hovick et al. 2012, Vercellino et al. 2021).

The performance of hybrids may also be influenced by maternal genetic effects (hereafter, maternal effects) (Kimball et al. 2008, Hernández et al. 2021), where the phenotype of the maternal parent affects offspring traits beyond the typical genetic inheritance (Roach and Wulff 1987). Although the ecological and evolutionary impacts of crop-to-wild/weed hybridization have been extensively studied (Campbell et al. 2006, 2009a, Mercer et al. 2006, 2007, Snow et al. 2010), the reverse hybridization process from wild/weed to crop, and its potential maternal effects have received considerably less attention (Kimball et al. 2008, Piskurewicz et al. 2016). If maternal effects exist, the performance of hybrids derived from crop and weed plants will differ. Therefore, common garden experiments comparing bidirectional crop–wild/weed hybrids and their respective parents across contrasting environments are key to advancing our understanding of how the hybridization processes influence weediness and invasiveness (Mercer et al. 2006, Campbell and Snow 2007).

Hybridization among divergent plant taxa, such as crop and wild/weeds relatives, can generate novel variation on which natural selection may act (Ellstrand et al. 2013). Selection typically acts on traits affecting fitness, and those correlated with them, shaping the phenotypic distribution within populations by influencing multiple fitness components (Kingsolver et al. 2001, Edelaar et al. 2023). This process can favour advantageous trait combinations within a given environment, potentially driving adaptive divergence in multivariate phenotypes between contrasting environments (Christe et al. 2016, Walter et al. 2018). Phenotypic selection analysis is an effective tool for estimating the strength and direction of linear and quadratic selection on functional quantitative traits. It provides key insights into how traits contribute to fitness in specific environmental contexts (Lande and Arnold 1983, Kingsolver et al. 2001, Caruso and Maherali 2020). This approach has advanced our understanding of selection dynamics in wild/weed and crop-wild/weed hybrids across diverse scenarios (Campbell et al. 2009a, Parachnowitsch and Kessler 2010, Emel et al. 2017, Exposito-Alonso et al. 2018, Presotto et al. 2019). For example, Presotto et al. (2019) observed that wild-like traits were favoured across a range of environments, whereas crop-like traits, particularly those related to rapid growth and fecundity, were predominantly selected in competitive environments. Campbell et al. (2009a) found that crop-weed hybrids exhibited larger size and stronger selection on size compared to weeds. Given the repeatability of evolutionary process following hybridization (Campbell et al. 2006, Stern 2013, Stankowski and Streisfeld 2015, Mitchell et al. 2022), field-based experimental hybridization studies offer valuable insights and allow for tentative predictions regarding the effects of crop-wild/weed hybridization on weed population dynamics and the potential for crop allele introgression into weed populations (Mercer et al. 2006, Campbell et al. 2009a, Mitchell et al. 2022).

The *Raphanus* genus, including the cultivated *R. sativus* L., its weedy conspecific forms, and the related species *R. raphanistrum* L., has become a well-established model system for studying the ecological and evolutionary impacts of crop–weed hybridization (Campbell et al. 2006, 2009a, Snow et al. 2010, Hovick et al. 2012, Shukla et al. 2021). For this complex, previous results showed that bidirectional crop-weed hybrids, regardless of the direction of hybridization, survive as much as their parents, with greater biomass and fecundity, resulting in higher fitness in both ruderal and agrestal environments. This suggests that intraspecific crop–weed hybridization has the potential to promote weediness in weedy *R. sativus,* particularly through increased plant size, which is commonly associated with enhanced competitive ability and crop interference (Vercellino et al. 2023a, 2023b). In South America, weedy *R. sativus* may exhibit reduced genetic variability due to their history of multiple demographic bottlenecks, including domestication, de-domestication, and introduction into South America (Vercellino et al. 2023a). Under this scenario, hybrids carrying crop alleles could experience increased phenotypic variance and potentially express trait combinations rare or absent in the weedy parental population. Because hybrids exhibited higher fitness in both environments (Vercellino et al. 2023b), stronger directional selection may occur if these new phenotypes lie farther from the current adaptive peak of weedy populations, as well as stronger quadratic selection if trait variances are greater than those observed in weedy populations. However, how selection acts on phenotypic traits, and how environmental context shapes patterns of selection, remains poorly understood. Although comparisons of selection estimates among different biotypes of the same species are relatively uncommon, such analyses can provide valuable insight into the evolutionary outcomes of their coexistence within the same environment (Campbell et al. 2009a, Presotto et al. 2019).

In this study, we analyse fecundity data previously reported in Vercellino et al. (2023b) alongside largely unexplored trait data from weeds and bidirectional first-generation crop–weed hybrids of *R. sativus*. Although first-generation hybrids do not fully determine the eventual fate of crop alleles, they strongly influence the initial trajectory of introgression by affecting the frequencies of crop alleles that are later subject to selection and drift (Snow et al. 1998, Mercer et al. 2006, Presotto et al. 2019). Our goal was to investigate the phenotypic variation of functional traits, and the selection operating on them, in the two contrasting environments where the populations occur. Specifically, we aimed to (1) assess the effects of environment (ruderal and agrestal), cross type (crop, weed, and bidirectional crop–weed hybrids), and their interaction on a set of functional traits, (2) evaluate phenotypic variation in these functional traits across cross types and environments, and (3) estimate direct linear and quadratic selection gradients acting on this set of traits, examining how selection differs among weeds and bidirectional crop-weed hybrids across environments. We hypothesized that crop-weed hybrids exhibit larger plant size in both environments and greater phenotypic variation than weedy conspecifics, and that selection on functional traits is stronger in crop-weed hybrids compared to their weedy parents, thereby promoting the evolution of weediness following hybridization. By gaining a deeper insight into the phenotypic variation in, and selection operating on, functional traits across these contrasting environments, we expanded our understanding of how differential selection influences the evolutionary trajectory of weediness following bidirectional crop–weed hybridization.

## Materials and methods

### Study system

Both cultivated and weedy radish (*Raphanus sativus*, Brassicaceae) are annual, self-incompatible, insect-pollinated species capable of hybridizing over long distances (Ellstrand et al. 1989, Snow and Campbell 2005). Cultivated radish, domesticated independently in Europe and Asia (Huh et al. 2024), was selected for multiple purposes, including human consumption as roots, forage and cover crops (Snow and Campbell 2005). Although *R. sativus* is unknown in the wild (Warwick 2011), it has naturally developed weedy (feral) populations in agricultural landscapes across diverse regions, including North and South America, Asia, and South Africa (Hegde et al. 2006, Barnaud et al. 2013, Vercellino et al. 2018, Huh et al. 2024). In North America, its invasiveness is attributed to introgression with the related species *R. raphanistrum,* commonly known as California wild radish (Hegde et al. 2006, Ellstrand et al. 2010, Zhang et al. 2021), and in South America, it is found across Brazil, Chile, Uruguay, Paraguay and Argentina (Theisen 2008, Pandolfo et al. 2018b, Oreja et al. 2024). In Argentina, weedy radish has been recognized as an invasive weed since at least the early 1900s (Vercellino et al. 2023a). It grows in disturbed habitats such as roadsides, rural roads, and fence lines and within agricultural fields. It is one of the most noxious weeds in cereal and oilseed crops (Scursoni et al. 2014, Oreja et al. 2024, Vercellino et al. 2024a) and has developed resistance to acetohydroxyacid synthase (AHAS, also known as acetolactate synthase [ALS])-inhibiting herbicides (Pandolfo et al. 2016). Argentine weedy radish populations have several crop-like traits such as white or purple flowers, mature pods attached to the mother plant, and lack of seed dormancy (Vercellino et al. 2019, Vercellino et al. 2024b), and they do not harbour typical traits of *R. raphanistrum,* such as constricted pods and yellow flowers (Campbell et al. 2006, Pandolfo et al. 2018b). In most areas severely infested with weedy radish (Vercellino et al. 2018, Oreja et al. 2024), the use of cultivated radish as a horticultural and cover crop has grown rapidly in recent years, leading to the formation of natural crop–weed hybrids.

### Germplasm and crosses

Weedy radish populations were collected from two sites in southern Buenos Aires: Balcarce (BAL; 37°35′25″S, 58°31′59″W) in 2008 (canola field) and Pieres (PIE; 38°24′31″S, 58°35′60″W) in 2011 (sunflower field) (Vercellino et al. 2018), prior to the introduction of *R. sativus* cover crop cultivars in those areas. Cultivated materials were represented by two commercial cultivars: Rovi, a European round red radish with white tips (Garde, Giusti y Chuchuy S.A., Ciudad Autónoma de Buenos Aires, Argentina) cultivated for root consumption; and CCS 779, a daikon radish (El Cencerro, Coronel Suarez, Buenos Aires, Argentina) cultivated as a cover crop. Radishes are cultivated in urban and peri-urban farms and gardens throughout the country, and as a cover crop in the Pampas region and northern Argentina, Paraguay, and Brazil (Snow and Campbell 2005). Bidirectional crosses were generated between weed populations (BAL and PIE) and cultivars (referred to as CROP), and the crosses (hereafter referred to as biotypes) were characterized according to their egg and pollen parents.

**Figure 1:**
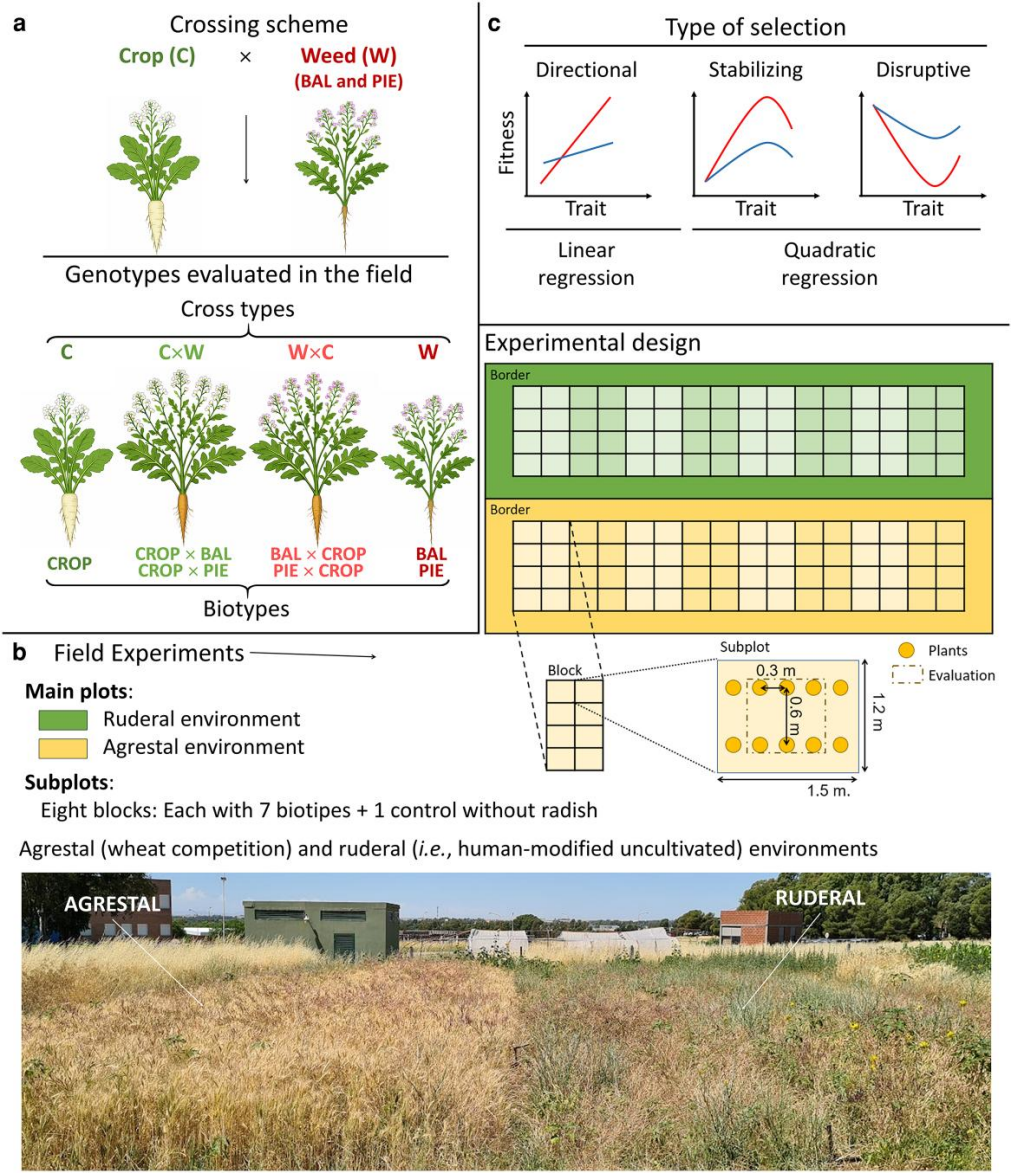
(a) Scheme of experimental crosses: Two weed populations (BAL and PIE) and the CROP were used to obtain seven biotypes (BAL, BAL × CROP, CROP × BAL, CROP, CROP × PIE, PIE × CROP, and PIE), which were grouped into four cross types: Weed (BAL and PIE), Weed × Crop (BAL × CROP and PIE × CROP), Crop × Weed (CROP × BAL and CROP × PIE), and Crop (CROP) each year. Crosses were evaluated in field experiments (b) Field experiments: Experimental design. Each environment comprised eight blocks, each containing eight subplots. Subplots included seven radish biotypes and a control plot without radish. (c) Types of selection inferred by linear and quadratic regression models for traits showing stronger (red) or weaker (blue) selection coefficients.

To minimize maternal environmental effects, we produced all seeds in a greenhouse (20 ± 5°C, natural light) at the Agronomy Department at Universidad Nacional del Sur, Bahía Blanca, Argentina. For seed production, 60 plants from each of the BAL, PIE, and CROP were grown and hand-pollinated, creating seven biotypes: three pure parents (BAL, PIE, and CROP) and four bidirectional F1 crop-weed hybrids (BAL × CROP, PIE × CROP, CROP × BAL, and CROP × PIE) (Fig. 1a), as we described in Vercellino et al. (2023). CROP was represented by the cultivar Rovi in 2019 and CCS 779 in 2020. For experiments conducted in 2020 and 2021 (hereafter Year 1 and Year 2, respectively), seeds were produced during the previous growing season, then stored at room temperature with moisture content below 10% until use.

### Site and experimental design

We compared weed, crop, and bidirectional hybrids in common garden experiments conducted in two environments (ruderal and agrestal) in the Agronomy Department experimental field at the Universidad Nacional del Sur, Bahía Blanca, Argentina (38°41′38″S, 62°14′53″W) during two winter growing seasons (the typical life cycle for the crop and weedy radish). The site has a semiarid, temperate climate, with an average annual temperature of 14.9°C. July is the coldest month (average 7.6°C) and January the warmest (average 23.0°C). Mean annual precipitation is 641 mm (SMN 2024, https://www.smn.gob.ar/). The soil has a well-drained loamy sand texture with 1.1% organic matter, pH 7.7, and 8.4 mg kg^−1^ (0–12 cm) available phosphorus (a typical soil in southwestern Buenos Aires Province).

Field experiments followed a split-plot design, with the two environments (agrestal and ruderal) as main plots (Fig. 1b). Each main plot was divided into eight blocks, and each block into eight subplots. The seven biotypes (CROP, BAL, PIE, CROP × BAL, CROP × PIE, BAL × CROP and PIE × CROP) and an empty control were randomly assigned to the subplots within each block, so that subplots were randomized for each block, environment, and year. Each subplot contained 10 plants of the same biotype. Subplots consisted of two rows spaced at 0.6 m, with five plants per row spaced at 0.3 m (Fig. 1b). Each main plot contained 64 subplots ([7 biotypes + 1 control without radish] × 8 blocks), totalling 1120 plants per experiment (7 biotypes × 8 blocks × 10 plants per subplot × 2 environments). The experiment was conducted over two successive years.

In the agrestal environment, wheat (*Triticum aestivum* L.) was planted before the establishment of the radish biotypes in late autumn (mid-June). Wheat was sown in rows spaced 0.2 m apart at a density of 160 plants m^−^² (a common stand density in southern Buenos Aires). The wheat cultivar ACA 360 was used in Year 1 and KLEIN MINERVA in Year 2. Wheat rows were arranged to surround, rather than overlap, the radish plants. Fertilization included 120 kg ha^−1^ diammonium phosphate at planting, followed by two applications of 125 kg ha^−1^ urea at early tillering and mid-tillering (*i.e*. early and mid-shoot/tiller formation), corresponding to Zadoks growth stages Z2.1 and Z2.4, respectively (Zadoks et al. 1974). Non-radish weeds were manually removed. At the end of each experiment, wheat yield was estimated by hand-harvesting aboveground biomass and threshing (*i.e.* separating grains from the remaining plant material) plants from four replicate 1 m^2^ areas in subplots without radish, averaging 5136 ± 201 in Year 1 and 5190 ± 188 kg ha^−1^ in Year 2. In the ruderal environment, no crops were planted, and the soil was not fertilized. Biotypes were exposed to natural conditions, including weed competition, herbivores, and pathogens, simulating a human-disturbed uncultivated area (*e.g.* field margin, fence row). To minimize border effects, each main plot was surrounded by two rows of weedy radish. Experiments were drip-irrigated, with initial supplemental watering to aid plant establishment, followed by additional watering as necessary. Further details provided in Vercellino et al. (2023b).

### Morphological trait measurements

For the six central plants per subplot, we recorded 16 traits at three plant stages: (1) Rosette stage (45 days after sowing): leaf length, leaf width, leaf area (estimated as leaf length × leaf width), rosette diameter, and leaf number; (2) Flowering stage: time to flowering (days from emergence to first flower), leaf length, leaf width, leaf area (as above), leaves number, plant height, and stem diameter. Trait values at the flowering stage were recorded on the day of first flowering. Leaf measurements were taken on the largest leaf, and only leaves longer than 1 cm were counted for leaf number; (3) Maturity stage: plant height, stem diameter, branch number, and root diameter. Plant fitness (*i.e.* seeds per plant and survival to reproduction) was analysed in a previous study (Vercellino et al. 2023b). The selected traits represent key functional dimensions associated with fitness at different life stages, encompassing early establishment, reproductive timing, and plant size and architecture related to resource acquisition and fecundity. Here, we used fecundity (*i.e.* number of seeds per plant; see Vercellino et al. 2023a) as the response variable in our selection analyses following the methods of Presotto et al. (2019).

### Statistical analyses

All data were analysed using generalized linear mixed models (GLMMs) with restricted maximum likelihood in PROC GLIMMIX (SAS University Edition, SAS Institute, Cary, NC, USA). The seven biotypes were grouped into four cross types: WEED (BAL and PIE), WEED × CROP (crop-weed hybrids produced on weed plants: BAL × CROP and PIE × CROP), CROP × WEED (crop-weed hybrids produced on crop plants: CROP × BAL and CROP × PIE), and CROP (Fig. 1a; see Vercellino et al. 2023a). First, we analysed the effects of experimental factors on trait values. For each year, environment (ruderal and agrestal), cross type, and their interaction were considered as fixed effects, while block nested within environment, biotype nested within cross type × environment, and individual plants (replicates) nested within environment × biotype × block were considered as random effects. The structure of the random effects was assessed using caterpillar plots (Matías and Jump 2015). Cross types were compared using Fisher’s LSD (*P* < .05). To reduce redundancy, we performed a multiple Pearson’s correlation analysis between traits using PROC CORR in SAS, and for trait pairs with *r* > |0.9|, we retained only one variable. This filtering resulted in 10 traits: rosette diameter (RSD), rosette leaf number (RLN), time to flowering (FT), leaf area at flowering (FLA), leaf number at flowering (FLN), plant height at flowering (FPH), stem diameter (FSD), plant height at maturity (MPH), branch number (BN) and root diameter (RTD) (see Supplementary Tables S1 and S2). The analysis of time to flowering included only radish plants that flowered, and trait analyses included only those that survived to reproduction. When necessary, data were square-root-transformed to meet the assumptions of normality (Mercer et al. 2006, Presotto et al. 2019). Differences in phenotypic variance among cross types for each trait, environment, and year were assessed using Levene’s test (α = 0.05). Because differences in variance detected by Levene’s test can be influenced by differences in trait means, we additionally quantified relative phenotypic variation using coefficients of variation (CV, %), calculated as the standard deviation divided by the mean, providing a descriptive measure of the magnitude of variation to aid biological interpretation. Levene’s test indicated statistical differences in variance, while CV provide context on the relative size of these differences. We also performed a principal component analysis (PCA) on the ten traits for each year, considering the combinations of environment and cross type. The PCA was conducted using the *prcomp()* function in R (R Core Team, 2025).

Second, we performed phenotypic selection analysis to estimate the strength and direction of direct selection acting on multiple traits (Lande and Arnold 1983, Dingemanse et al. 2021, Edelaar et al. 2023). Linear and quadratic regressions were employed to estimate selection coefficients based on the relationship between fecundity and trait values using a Generalized Linear Mixed Model (GLMM) analysis in PROC GLIMMIX. Fecundity (number of seeds per plant) was evaluated in a previous study (Vercellino et al. 2023b). Here, we additionally included individuals with zero fecundity that had not survived to reproduction, previously excluded. We estimated linear (β) and quadratic (γ) selection gradients, following the methods described by Presotto et al. (2019). Selection gradients represent direct selection. The linear selection coefficient estimate linear selection and indicate whether selection favors larger or smaller trait values, while the quadratic coefficient estimate curvature in the trait–fitness relationship (Fig. 1c) (Lande and Arnold 1983, Kingsolver et al. 2001, Dingemanse et al. 2021). Analysis of quadratic selection can help identify potential stabilizing (negative quadratic coefficients) or disruptive (positive quadratic coefficients) selection (Fig. 1c) (Parachnowitsch and Kessler 2010, Presotto et al. 2019); though their interpretation can be complex (Shaw and Geyer 2010). To obtain appropriate coefficients from the quadratic regression model, we doubled the quadratic selection coefficients and their standard errors (Stinchcombe et al. 2008). All traits were standardized to a mean of 0 and a standard deviation of 1 to allow direct comparison of the strength, direction and shape of selection across traits (Wolfe and Tonsor 2014) and fecundity was relativized (all values divided by the mean resulting in a mean of one). We excluded the CROP cross type, given its low likelihood of reverting to a feral biotype within a few generations (Campbell and Snow 2009), making it irrelevant for selection analysis. In addition, its low survival rate in Year 1 (Vercellino et al. 2023b) made the analysis challenging (Kingsolver et al. 2001).

The basic model for linear and quadratic selection analyses included, for each year: environment (ruderal and agrestal), cross type (WEED, WEED × CROP, and CROP × WEED), and their interaction (environment × cross type) as fixed effects, with block nested within environment and biotype nested within cross type × environment as random effects (De Lisle and Svensson 2017). This mixed model was used to analysed the effects of experimental factors (environment, cross type, and the environment by cross type interaction). When significant trait by environment or trait by cross type interactions were detected, selection analyses were conducted separately within each environment and cross type, respectively. When a significant trait by environment by cross type interaction was detected, selection analyses were conducted separately for each combination of environment and cross type. Linear (*β′*) and quadratic (*γ′*) selection gradients were estimated using standard multiple regression of relative fitness on standardized traits (Lande and Arnold 1983). Linear selection gradients (*β*′) were estimated from linear-only models, whereas quadratic selection gradients (*γ*′) were estimated from models including both linear and quadratic terms.

## Results

### Effects of environment, cross type, and its interactions on trait values

In both years, most of the ten functional traits exhibited significant effects of environment and cross type, with environment by cross-type interactions largely non-significant or weak (Table 1; see Supplementary Tables S3 and S4), indicating broadly similar responses of cross types across environments.

**Table 1:** Restricted maximum likelihood for ten traits: Rosette diameter (RSD), rosette leaf number (RLN), time to flowering (FT), leaf area at flowering (FLA), leaf number at flowering (FLN), plant height at flowering (FPH), stem diameter at flowering (FSD), plant height at maturity (MPH), branch number (BN), and root diameter (RTD), evaluated in four cross types: bidirectional crop–weed hybrids and their progenitors (weeds and crop) of Raphanus sativus, in two environments (ruderal and agrestal) using maximum likelihood in SAS PROC GLIMMIX in two years.

Traits	Year 1			Year 2		
	E	CT	E*CT	E	CT	E*CT
RSD	109.72***	12.45**	0.50 ns	208.31***	26.08***	4.62 ns
RLN	67.81***	12.38**	3.36 ns	11.22*	9.07*	2.62 ns
FT	12.70*	48.22***	3.65 ns	2.61 ns	0.50 ns	0.15 ns
FLA	90.97***	20.36**	0.99 ns	216.48***	40.80***	10.66**
FLN	99.38***	26.97***	10.13**	16.26**	126.81***	1.33 ns
FPH	283.62***	51.28***	1.60 ns	486.54***	12.52**	2.02 ns
FSD	83.18***	30.39***	4.16 ns	94.15***	44.83***	5.71*
MPH	177.19***	42.02***	7.75*	494.68***	38.61***	3.19 ns
BN	69.94***	36.36***	4.72 ns	7.56*	77.88***	2.92 ns
RTD	17.56**	92.76***	0.33 ns	0.06 ns	41.63***	1.25 ns

Data were collected in the Agronomy Department at Universidad Nacional del Sur, Bahía Blanca, Argentina. For each Year, the model included environment, cross type, and their interaction (environment × cross type) as fixed effects, and block nested within environment, biotype nested within cross type × environment, and individual plants within environment × biotype × block as random effects. Numerator and denominator degrees of freedom and random effects are in Supplementary Tables S3 and S4.

Numbers refer to *F* value.

Symbols indicate the significance of environment (E), cross type (CT), and environment by cross type (E*CT): ns (non-significant). *P < .05. **P < .01. ***P < .001.

**Figure 2:**
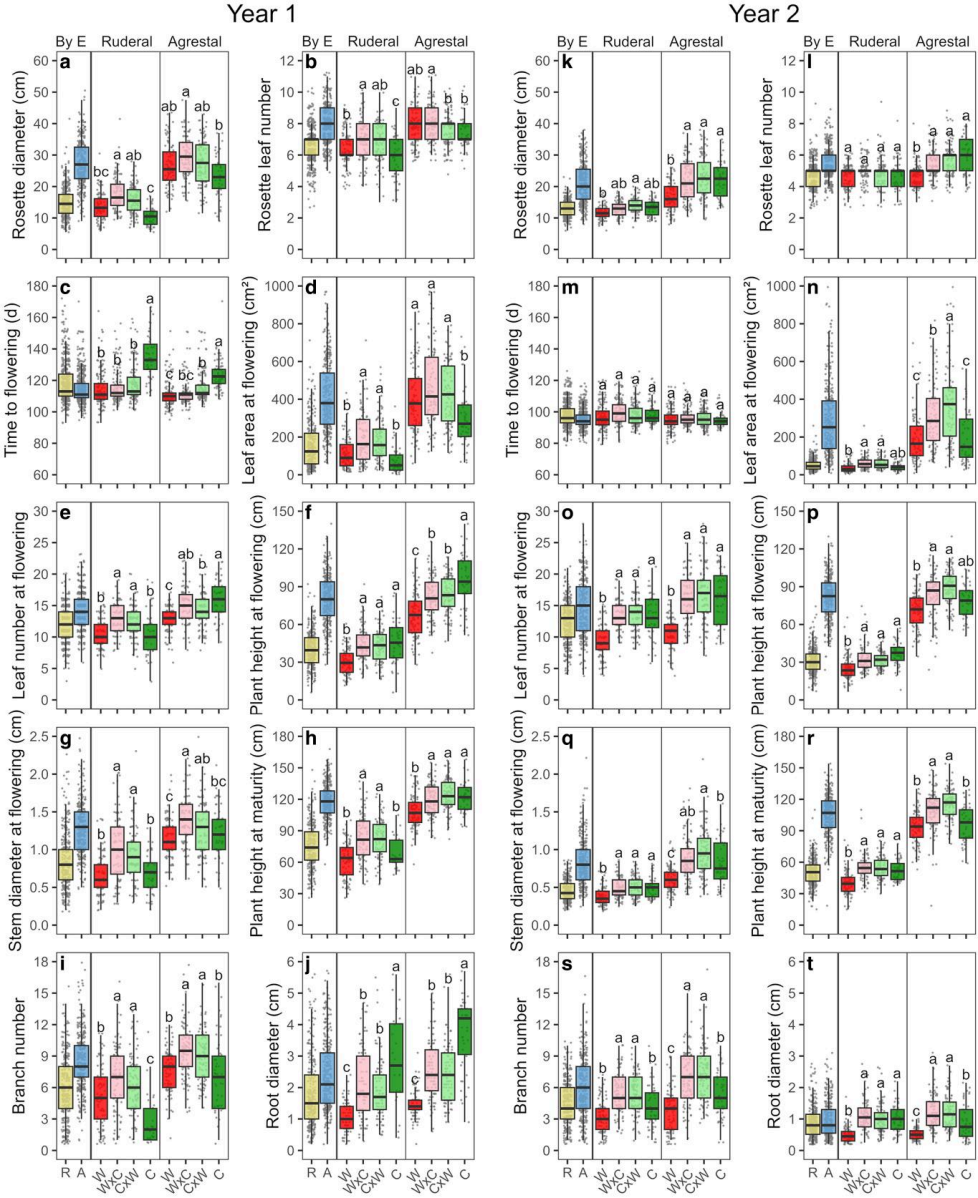
Boxplots of rosette diameter (RSD), rosette leaf number (RLN), time to flowering (FT), leaf area at flowering (FLA), leaf number at flowering (FLN), plant height at flowering (FPH), stem diameter at flowering (FSD), plant height at maturity (MPH), branch number (BN) and root diameter (RTD), in four cross types (CT): crop (C), weed (W), and bidirectional crop-weed hybrids (W × C and C × W) of radish (*Raphanus sativus* L.) across two contrasting environments (E): Ruderal (R) and agrestal (A), over two years (Year 1 and 2). Box edges represent the 0.25 and 0.75 quartiles, solid line represents the median value, and whiskers extend to the Tukey value. Transparent points represent each data point. Values sharing the same letter within an environment and year are not significantly different based on Fisher LSD tests (*P* > .05), and different letters within a panel indicate significant differences as determined by the Fisher’s LSD tests (*P* < .05).

Plants in the agrestal environment were larger across rosette, flowering, and maturity stages, with greater root diameter (except in Year 2), than those in the ruderal environment. Flowering time differed slightly between environments only in Year 1 (<4 days), with no differences detected in Year 2 (Fig. 2, Table 1).

Most functional traits differed significantly between parental cross types (crop and weeds) across environments (Fig. 2). In Year 1, the crop had higher values than weeds for flowering time, plant height at flowering, and root diameter, while other traits showed minimal or non-significant differences. Some traits exhibited environment-specific responses, with higher values in weeds or in the crop depending on the environment (Fig. 2a–j). In Year 2, the crop generally exhibited higher values than weeds for rosette diameter, leaf number and stem diameter at flowering, plant height, branch number, and root diameter, with minimal or no differences between the crop and weeds for rosette leaf number, flowering time and leaf area at flowering (Fig. 2k–t).

Overall, bidirectional hybrids exhibited comparable phenotypic values across environments and years, suggesting no maternal genetic effects (Fig. 2). Across both environments and years, hybrids exhibited most functional traits at levels equal to or higher than those of both parents (Fig. 2). Compared to weeds, hybrids in Year 1 exhibited significantly greater values for most flowering and maturity traits, and root diameter. Rosette leaf number and flowering time were similar between hybrids and weeds. In Year 2, hybrids had significantly higher values than weeds for most traits at the rosette, flowering, and maturity stages and for root diameter across both environments, while rosette leaf number and flowering time were similar. Compared to the crop, in Year 1, hybrids generally showed significantly higher values for most rosette and flowering traits and branch number, but lower flowering time, plant height at flowering and root diameter. In year 2, hybrids generally showed similar values to the crop for most rosette and flowering traits, while exhibiting higher leaf area at flowering and branch, as well as greater plant height at maturity and root diameter in the agrestal environment, with no significant differences in the ruderal environment.

Overall, differences in phenotypic variation among cross types were limited. Although Levene’s test revealed significant differences in phenotypic variance among cross types for multiple traits, with patterns varying depending on environment and year (see Supplementary Table S5), crop–weed hybrids generally exhibited coefficients of variation (CV) that were intermediate or similar to those of their parental lines, suggesting minimal differences among cross types in phenotypic variation (Supplementary Table S6).

**Figure 3:**
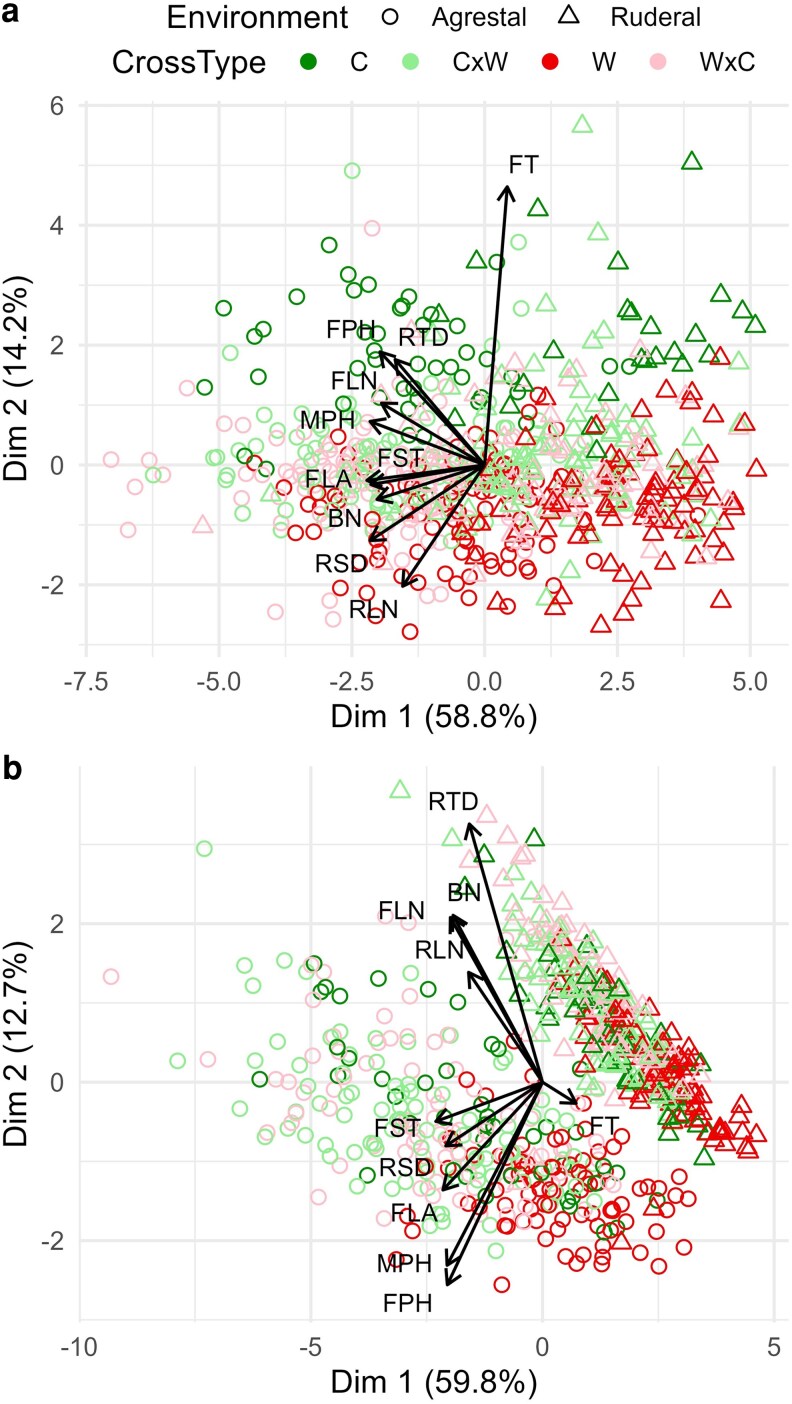
Principal component analysis (PCA) for rosette diameter (RSD), rosette leaf number (RLN), time to flowering (FT), leaf area at flowering (FLA), leaf number at flowering (FLN), plant height at flowering (FPH), stem diameter at flowering (FSD), plant height at maturity (MPH), branch number (BN) and root diameter (RTD), in four cross types: crop (C), weed (W) and bidirectional crop-weed hybrids (W × C and C × W) of radish (*Raphanus sativus* L.) across two contrasting environments: Ruderal (triangle) and agrestal (circle), over two years: Year 1 (A) and Year 2 (B).

In the PCA, the first two dimensions explained 73.0% of the total phenotypic variance in Year 1% and 72.5% in Year 2, discriminating both environments and cross types (Fig. 3). In Year 1, weeds were clearly separated from crops, with hybrids occupying intermediate positions, whereas in Year 2 hybrids were phenotypically distinct from both parental types (Fig. 3).

### Phenotypic selection analysis

**Figure 4:**
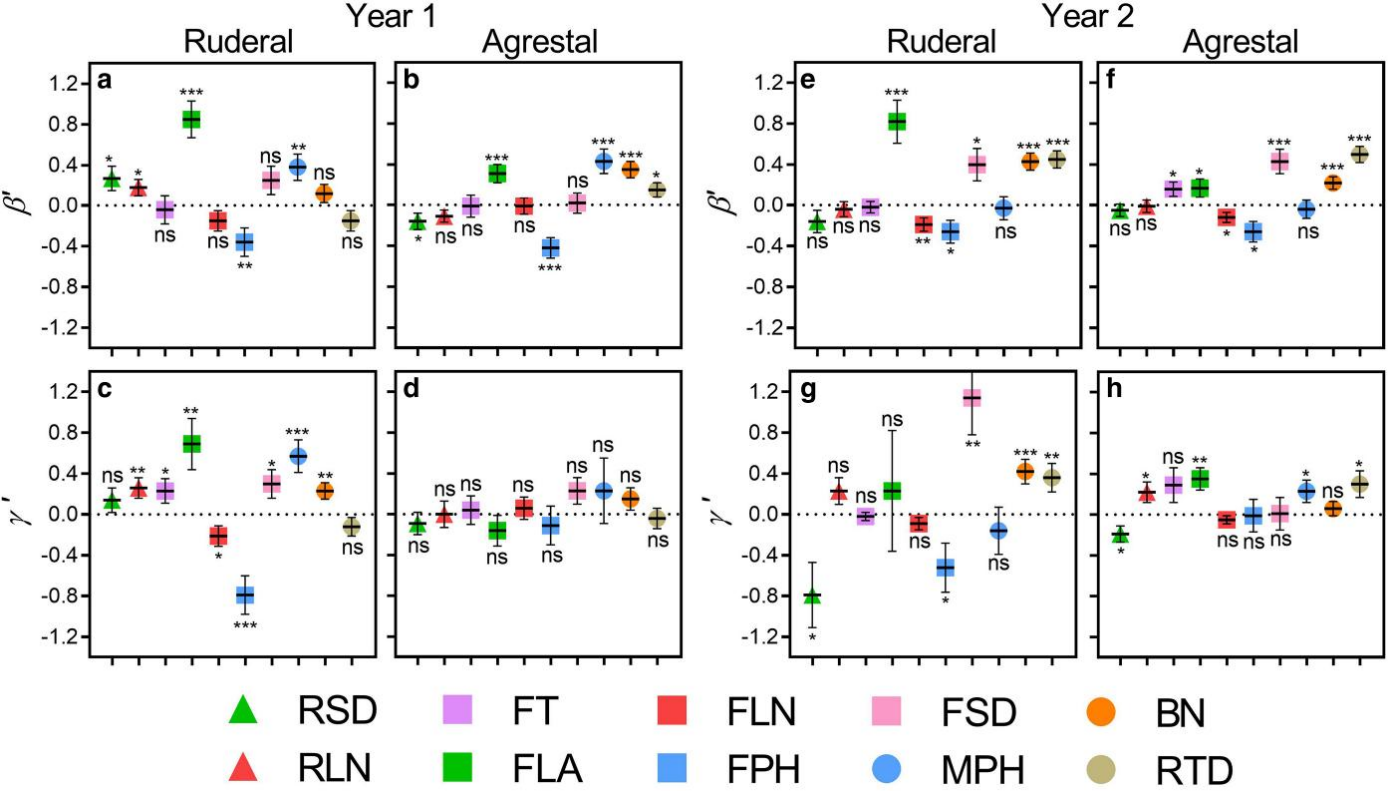
Linear (*β*′) and quadratic (*γ*′) selection gradients, for ten traits across three plant stages. Seedling stage (triangle): rosette diameter (RSD) and rosette leaf number (RLN); flowering stage (square): time to flowering (FT), leaf area at flowering (FLA), leaf number at flowering (FLN), plant height at flowering (FPH), and stem diameter at flowering (FSD); maturity stage (circle): plant height at maturity (MPH), branch number (BN), and root diameter (RTD), in weed and bidirectional crop-weed hybrids of radish (*Raphanus sativus* L.) across two contrasting environments (Ruderal and Agrestal) over two years (Year 1 and 2). Data are shown by environment: Ruderal and Agrestal. The horizontal line represents the mean, and the vertical lines represent ± SE. Symbols indicate the significance of selection estimators: ns (non-significant); * *P* < .05; ** *P* < .01; *** *P* < .001.

**Figure 5:**
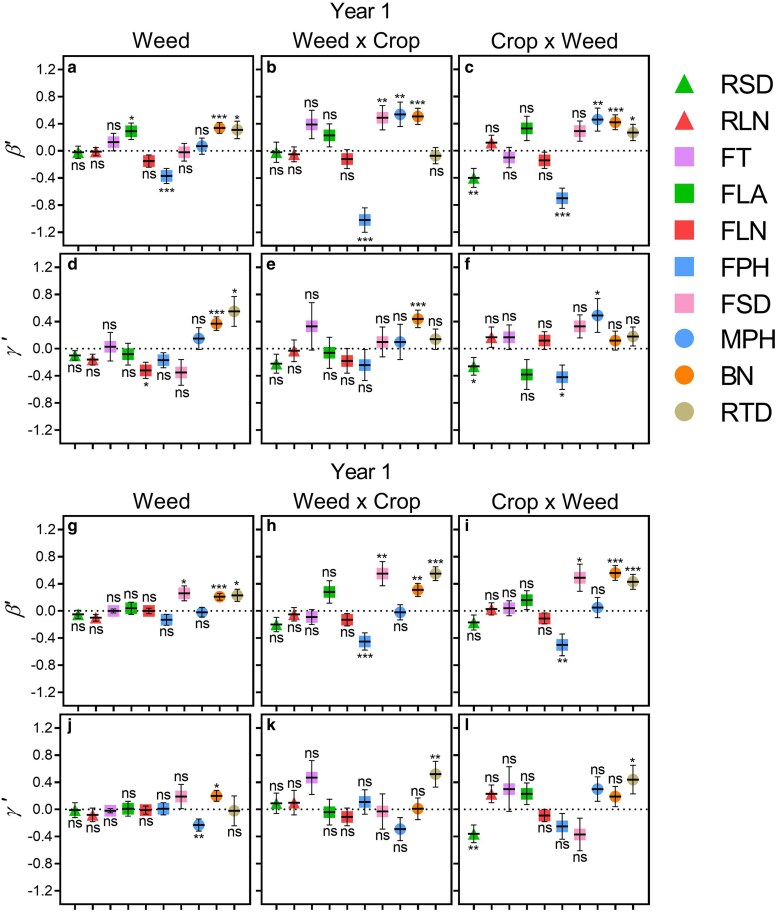
Linear (*β*′) and quadratic (*γ*′) selection gradients, for ten traits across three plant stages. Seedling stage (triangle): rosette diameter (RSD) and rosette leaf number (RLN); flowering stage (square): time to flowering (FT), leaf area at flowering (FLA), leaf number at flowering (FLN), plant height at flowering (FPH), and stem diameter at flowering (FSD); maturity stage (circle): plant height at maturity (MPH), branch number (BN), and root diameter (RTD), in weed and bidirectional crop-weed hybrids of radish (*Raphanus sativus* L.) across two contrasting environments (ruderal and agrestal) over two years. Data are shown by cross type: WEED, WEED × CROP, and CROP × WEED. The horizontal line represents the mean, and the vertical lines represent ± SE. Symbols indicate the significance of selection estimators: ns (non-significant); * *P* < .05; ** *P* < .01; *** *P* < .001

Due to the low survival rate of the crop in Year 1 and the resulting imbalance in the data, combined with its limited potential to revert to a feral biotype (Campbell and Snow 2009), the CROP was excluded from the phenotypic selection analysis. We thus evaluated linear and quadratic selection gradients for the remaining cross types (Weed, Weed × Crop hybrids, and Crop × Weed hybrids) across two environments (Table 2). Given our focus on the phenotypic selection analysis across environments and cross types, and the limited biotype × environment interactions (Table 2, see Supplementary Tables S7–S10), we presented the selection estimators (*β*′ and *γ*′) by environment (Fig. 4) and cross type (Fig. 5).

**Table 2:** Restricted maximum likelihood for direct linear (β′) and quadratic (γ′) selection, of ten traits (T): Rosette diameter (RSD), rosette leaf number (RLN), time to flowering (FT), leaf area at flowering (FLA), leaf number at flowering (FLN), plant height at flowering (FPH), stem diameter at flowering (FSD), plant height at maturity (MPH), branch number (BN), and root diameter (RTD), evaluated in three cross types (CT, weed and bidirectional crop–weed bidirectional hybrids) of Raphanus sativus, in two environments (E, ruderal and agrestal) across two years (Year 1 and 2).

Year 1	β′				γ′			
Traits (T)	T	T*E	T*CT	T*E*CT	T	T*E	T*CT	T*E*CT
RSD	0.51 ns	7.15**	1.83 ns	1.73 ns	0.79 ns	3.08 ns	4.23*	5.80**
RLN	0.42 ns	12.59***	0.19 ns	0.16 ns	7.26**	9.00**	0.30 ns	0.25 ns
FT	0.04 ns	0.79 ns	1.09 ns	0.31 ns	0.35 ns	1.60 ns	0.11 ns	1.22 ns
FLA	20.74***	5.31*	0.01 ns	0.23 ns	8.47**	12.61***	1.68 ns	1.44 ns
FLN	1.61 ns	2.64 ns	0.31 ns	0.55 ns	9.90**	3.96*	2.13 ns	4.18*
FPH	26.55***	0.32 ns	1.32 ns	1.96 ns	8.82**	1.39 ns	0.73 ns	0.93 ns
FSD	1.87 ns	2.24 ns	2.34 ns	0.13 ns	43.66***	33.48***	0.93 ns	1.12 ns
MPH	14.56***	0.90 ns	1.61 ns	2.03 ns	0.72 ns	5.67*	3.24*	1.65 ns
BN	17.66***	0.65 ns	0.10 ns	0.25 ns	3.86 ns	3.84 ns	0.41 ns	0.08 ns
RTD	0.83 ns	3.63 ns	3.93*	0.92 ns	0.44 ns	2.14 ns	0.53 ns	0.41 ns

Year 2	β′				γ′			
Traits (T)	T	T*E	T*CT	T*E*CT	T	T*E	T*CT	T*E*CT
RSD	5.28*	1.55 ns	0.78 ns	1.37 ns	2.02 ns	7.01**	0.24 ns	1.56 ns
RLN	0.26 ns	0.99 ns	0.43 ns	0.54 ns	0.04 ns	0.42 ns	0.14 ns	1.49 ns
FT	0.52 ns	13.83***	0.72 ns	1.24 ns	8.28**	6.87**	3.79*	2.38 ns
FLA	15.19***	3.55 ns	0.21 ns	0.73 ns	10.06**	47.45***	3.43*	3.79*
FLN	9.40**	0.26 ns	0.53 ns	0.54 ns	3.28*	0.50 ns	0.56 ns	0.34 ns
FPH	18.08***	<0.01 ns	2.97 ns	0.19 ns	0.61 ns	2.87 ns	0.37 ns	0.83 ns
FSD	14.76***	<0.01 ns	0.77 ns	0.58 ns	0.79 ns	4.35*	0.50 ns	1.92 ns
MPH	<0.01 ns	0.03 ns	1.04 ns	2.34 ns	7.71**	6.09*	1.48 ns	2.35 ns
BN	50.05***	4.74*	3.34*	0.25 ns	0.11 ns	0.77 ns	1.63 ns	0.26 ns
RTD	40.03***	1.77 ns	0.67 ns	2.55 ns	0.04 ns	5.29*	2.22 ns	0.82 ns

Data were collected in the Agronomy Department at Universidad Nacional del Sur, Bahía Blanca, Argentina. For each Year, the model included trait (T), T × Environment (E), T × Cross type (CT), and their interaction (T × E × CT) as fixed effects, and block nested within environment, biotype nested within cross type × environment, and individual plants (replicates) nested within environment × biotype × block as random effects. Numerator and denominator degrees of freedom and random effects are in Supplementary Tables S7–S10.

Numbers refer to F value.

Symbols indicate the significance of trait (T), trait by environment (T*E), trait by cross type (T*CT), and trait by environment by cross type (T*E*CT): ns (non-significant), *P < .05. **P < .01. ***P < .001.

When evaluating directional selection gradients (*β*′), no significant interaction between environment and cross type was detected for any traits in either year (Table 2, Supplementary Tables S7 and 8), suggesting similar direct linear selection patterns across environments and cross types. Thus, *β*′ values were compared across environments and among cross type. All traits exhibited significant *β*′ in at least one environment or cross type combinations (Figs. 4 and 5). In Year 1, flowering leaf area, plant height, and branch number exhibited the strongest direct selection (Figs. 4 and 5), while in Year 2, leaf area, plant height, and stem diameter at flowering, branch number, and root diameter exhibited the strongest direct selection (Figs. 4 and 5). Flowering plant height was the only trait negatively selected in both years (Figs. 4 and 5). Environmental differences in *β*′ were generally limited, with only three traits in Year 1 and two traits in Year 2 showing significant effects in each year, without a consistent pattern (Table 2, Fig. 4). In Year 1, rosette diameter and leaf number, and leaf area at flowering exhibited stronger selection in the ruderal than in the agrestal environment (Fig. 4a–d), and in Year 2, branches number showed the same pattern (Fig. 4e–h). In both years, most traits (all except one) showed non-significant differences in *β*′ among cross types (Table 2), suggesting largely similar patterns of direct linear selection on functional traits among cross types; however, *β*′ values tended to be higher in hybrids than in weeds (Fig. 5a–c and g–i).

Regarding quadratic selection gradients (*γ′*), all traits exhibited significant *γ′* in some combination of environment or cross type across both years (Figs. 4 and 5). We found significant differences in *γ′* between environments for five of ten traits in Year 1 and six of ten traits in Year 2 (Table 2, Supplementary Tables S9 and 10). *γ*′ was generally stronger in the ruderal than in the agrestal environment (Fig. 4). In year 1, eight of ten traits showed significant *γ′* in the ruderal environment, with flowering leaf number and plant height showing negative *γ′*, whereas no trait exhibited significant *γ′*_ii_ in the agrestal environment (Fig. 4c and d). In contrast, in Year 2, rosette diameter and root diameter were negatively and positively selected, respectively, in both environments, while other traits selected in the ruderal environment were not selected in the agrestal one, and *vice versa* (Fig. 4g and h). On the other hand, *γ′* showed no significant differences among cross types for most traits (Fig. 5d–f and j–l, Table 2), suggesting similar patterns of direct quadratic selection on functional traits among cross types, although *γ′* values tended to be higher in hybrids in Year 2. In addition, there was generally no significant interaction between environment and cross type for most traits across both years (Table 2, Supplementary Tables S9 and 10). Although some significant differences were observed in specific combinations of environment and cross type, these did not substantially contribute to overall quadratic selection (Supplementary Tables S11 and 12).

## Discussion

Our findings revealed that bidirectional crop-weed hybrids consistently exhibited larger values for most functional traits compared to both parental types, regardless of hybridization direction, highlighting the potential for increased weediness following intraspecific crop-weed hybridization in weedy *R. sativus*. We observed both linear and quadratic selection acting on most traits, suggesting that natural selection acts simultaneously on multiple traits (Dingemanse et al. 2021). Overall, linear selection gradients (*β*′) were stronger for leaf area, plant height at flowering, and branch number, with plant height at flowering as the only trait negatively selected, indicating direct linear selection acting on this set of traits. Minimal differences in *β*′ between environments and cross types suggest similar linear selection across environments and among cross types, although linear selection tended to be higher in hybrids than in weeds. Quadratic selection (*γ′*) was generally stronger in the ruderal environment, with some traits showing negative and others positive values, indicating a complex pattern of selection and a challenging interpretation. This effect was also found by Presotto et al. (2019) in sunflower. Similar quadratic selection among cross types indicates generally comparable selection between hybrids and weeds, although quadratic selection tended to be stronger in hybrids.

It is possible that the strong positive direct linear selection for plant size (*e.g.* leaf area and stem diameter at flowering, and branch number) may result in reduced or no selection for flowering time in our environments (reviewed in Austen et al. 2017), where environmental conditions allow radish plants to flower for a large part of the year (Pandolfo et al. 2018b, Vercellino et al. 2023a, Oreja et al. 2024). Consistent with our results, Campbell et al. (2009a) reported that strong selection on plant size and phenotypic correlations between age at reproduction and plant size may have limited the response of flowering phenology in interspecific crop-weed radish. Similar patterns were observed in experiments selecting radishes directly for flowering time (Campbell et al. 2009b, Ashworth et al. 2016). These findings indicate that selection operates in a multivariate manner, with evolutionary changes depending on trait combinations expressed at different ontogenetic stages, thereby promoting coordinated patterns of adaptation. Understanding the interplay among functional traits and their impact on the expression of a higher-level fitness component (*i.e.* seed production) further highlights the importance of direct selection (Kingsolver et al. 2012, Caruso and Maherali 2020).

### Effects of environment and cross type on functional traits

The evaluation of the effects of bidirectional crop–weed hybridization under contrasting ecological conditions allowed us to assess the environmental dependence of hybridization outcomes. We found a generally consistent pattern for most traits across environments, cross types, and years. Overall, radish plants in the agrestal environment exhibited larger size at seedling, flowering, and mature stages compared, consistent with previous findings showing higher biomass and seed production in the agrestal relative to the ruderal environment (Vercellino et al. 2023b). This could be associated with increased resource availability in the agrestal environment (*e.g.* more nutrients due to fertilization, and more water due to lower direct evaporation) compared to the ruderal environment (Vercellino et al. 2023b).

When comparing cross types, we observed some differences between years, possibly linked to the origin of the cultivars: a European horticultural cultivar was used in Year 1, while an Asian cover crop cultivar was used in Year 2 (Snow and Campbell 2005, Huh et al. 2024). In Year 1, weeds and the crop exhibited similar plant size, while the crop flowered later and outperformed weeds in plant height at flowering and root diameter; traits strongly selected in horticultural radish cultivars (Snow and Campbell 2005, Charbonneau et al. 2018). In contrast, in Year 2, the crop outperformed weeds in most traits related to plant size (*e.g.* rosette diameter, rosette leaf number, leaf number, plant height and stem diameter at flowering, plant height at maturity, branch number, and root diameter), while flowering time and leaf area at flowering were similar, likely reflecting selection for larger size in cover crop or forage cultivars (Snow and Campbell 2005).

Most hybrids exhibited larger values in functional traits associated with plant size compared to weeds (*e.g.* leaf area, stem diameter, leaf number and plant height at flowering, plant height at maturity, branch number, and root diameter) across both environments and years, with minimal differences between bidirectional hybrids. This lack of maternal genetic effects contrasts with that reported by Hernández et al. (2021) in sunflower. Increased plant size in crop-weed hybrids relative to the weed parent has also been observed in interspecific hybrids between *R. sativus* and *R. raphanistrum* (Hegde et al. 2006). These findings are consistent with previous studies reporting increased fitness in crop-weed hybrids relative to their parents in radish (Campbell et al. 2009a, Vercellino et al. 2023b) and other taxa (Mitchell et al. 2022). Contrary to expectations, phenotypic variation in crop–weed hybrids remained similar to that of the parental lines. Although hybrids exhibited higher mean values in traits related to plant size, the distribution of trait values within the population was largely unchanged, suggesting that hybridization may not expand the phenotypic space available for selection (see below). This contrasts with previous reports of increased phenotypic diversity in interspecific *Raphanus* hybrids (Campbell et al. 2009a). Further research is needed to elucidate the long-term effects of intraspecific hybridization events (Campbell et al. 2009a, Snow et al. 2010, Mitchell et al. 2022).

### Selection of functional traits

Selection operates on functional traits through their combined effects, driven by direct selection. The direct impact of selection on specific traits was especially relevant for those related to plant size (*e.g.* leaf area at flowering, branches number). The evaluation of linear and quadratic selection gradients in weedy radish and its bidirectional crop–weed hybrids across contrasting ecological environments was conducted to predict the evolutionary outcomes of hybridization and to explore the environmental dependence of the selection process (Campbell and Snow 2007, Presotto et al. 2019). Weedy radish is widespread in both agrestal and ruderal environments across South America’s agricultural regions (Vercellino et al. 2018, Vercellino et al. 2023a, Oreja et al. 2024). In southern Buenos Aires (Argentina), a region severely infested with weedy radish (Scursoni et al. 2014, Vercellino et al. 2018, Oreja et al. 2024), agricultural environments are characterized by winter and summer crops under a no-till system, proper fertilization, water management, and chemical weed control, while ruderal environments, such as roadsides and field edges, are not fertilized, and weeds and pests are not controlled (Scursoni et al. 2019, Oreja et al. 2024). Given that direct linear selection (*β*′) favoured individuals with larger size (*e.g.* larger leaf area at flowering, taller plants at maturity, more branches, larger root diameter) in both ruderal and agrestal environments, our findings suggest that both environments exert sufficient linear selection on functional traits to produce rapid microevolutionary change in weedy radish, regardless of hybridization, as observed in other crop-wild complexes (Kingsolver and Diamond 2011, DiVittorio et al. 2021, Mitchell et al. 2022). The minimal environmental differences observed here are likely due to the adaptation of weedy radish to both environments, coupled with the relatively few phenotypic differences between crops and weeds in this complex, especially when compared to other crop-wild/weedy complexes, such as sunflower (Mercer et al. 2006, Casquero et al. 2013), corn (Le Corre et al. 2020), rice (Vigueira et al. 2019, Presotto et al. 2024), and sugar beet (Bartsch 2010).

Direct quadratic selection gradients (*γ*′) revealed that different traits were negatively or positively selected, especially in the ruderal environment, with no consistent pattern across years, hindering their interpretation. However, given that *R. sativus* is an obligately cross-pollinating, self-incompatible species, selection may help maintain phenotypic diversity of weedy radish in cultivated fields, aiding its adaptation to various control practices currently used in agricultural settings (Scursoni et al. 2019). For instance, agricultural practices not evaluated here, such as herbicide application and seed destruction during harvest (Walsh et al. 2018), could produce strong selection in the agrestal environment, favouring herbicide-resistant biotypes (Pandolfo et al. 2016, Vercellino et al. 2018) or those with shorter life cycles or smaller plants, better suited to overcoming agricultural challenges (Somerville and Ashworth 2024). This may have facilitated its adaptation to the diverse agricultural environments in our country (Oreja et al. 2024, AAPRESID 2025).

Contrary to our expectations, we observed few differences between cross types for both linear and quadratic selection, suggesting similar selection patterns with no clear advantage for any cross type. However, there was a tendency for stronger linear and quadratic selection in bidirectional hybrids compared to weeds, which could still promote greater evolutionary responsiveness of hybrids under changing environmental conditions, as stronger selection could translate into more pronounced trait shifts across generations, as even weak selection can produce incremental trait shifts across generations. This tendency may be associated with the larger plant size of hybrids or with crop alleles generating trait combinations that are rare or absent in the weedy parental population. Furthermore, the relatively weaker quadratic selection suggests that linear selection is likely the primary force driving the evolution of weedy radish, as seen in other species (Kingsolver et al. 2001, 2012). Notably, weeds also exhibited significant selection gradients, indicating that selection has been acting consistently across multiple generations (Campbell et al. 2009a). Given the minimal differences among cross types, we expect that advanced hybrid generations may retain enhanced phenotypic traits and higher fitness, thereby promoting the evolution of weedy radish following intraspecific crop–weed hybridization. This pattern has been observed in interspecific crop-wild/weedy hybrids in radish (Campbell et al. 2006, 2009a, Hegde et al. 2006, Campbell and Snow 2007, Hovick et al. 2012) and sunflower (Mitchell et al. 2022).

Flowering time is a key trait in the radish complex, known to be under strong selection (Campbell et al. 2009a, 2009b, Ashworth et al. 2016, Zhang et al. 2021). Early flowering leads to reduced biomass accumulation and fewer reproductive branches, resulting in lower fecundity, while late flowering promotes greater biomass and branch development, leading to higher fecundity (Campbell and Snow 2007, Campbell et al. 2009b, Ashworth et al. 2016). However, we did not detect direct linear selection (*β*′) on this trait, suggesting that flowering time may not be under strong selection. Consistent with this, Shukla et al. (2020) reported that flowering time in *R. raphanistrum* and hybrids between *R. sativus* and *R. raphanistrum* remained stable under drought-induced stress. Similarly, Campbell et al. (2009a) found strong selection on plant size, with a limited response in flowering time, in interspecific crop-weed radish hybrids. Nonetheless, flowering time could still be influenced by agricultural practices that exert strong selection, such as chemical control and seed harvest control (Ashworth et al. 2016, Somerville and Ashworth 2024).

Numerous studies have highlighted the interannual variation in the magnitude of selection (Kingsolver and Diamond 2011, Kingsolver et al. 2012, Wadgymar et al. 2017). In our study, however, linear and quadratic selection patterns remained relatively consistent across both years and radish cultivars, suggesting that selection acted on similar set of traits. This finding is consistent with Campbell et al. (2009), who found that selection operated on the same set of traits across multiple generations. Additionally, the magnitude of selection observed here was slightly higher than that reported in other weedy radish studies (Scheiner et al. 2002, Campbell et al. 2009a).

To our knowledge, this is the first study to estimate direct linear and quadratic selection coefficients in weedy and bidirectional crop-weed hybrids of radish. Our approach is particularly noteworthy because, through hybridization, we created phenotypes that are highly plausible in natural settings, given the recent large-scale introduction of cultivated radish as a cover crop in extensive areas of Argentina and neighbouring countries (Bertolotto and Marzetti 2017). This methodology is valuable as it accelerates the natural evolutionary process, allowing us to observe potential outcomes of bidirectional crop-weed hybridization. Our results suggest that weedy radish populations may benefit from bidirectional crop-weed hybridization and the acquisition of traits associated with increased size. Finally, a possible limitation of our common garden experiments is that hybrid fitness could be influenced by factors not assessed in our study, such as seed dormancy and seedling emergence (Mercer et al. 2014, Mitchell et al. 2022). However, fecundity remains the primary factor influencing hybrid population growth in the *Raphanus* complex (Campbell et al. 2014, 2016, Teitel et al. 2016). Future studies incorporating additional life-history traits and advanced-generation hybrids will be crucial to further validate our findings.

## Conclusion

Crop–weed hybridization in weedy radish has the potential to increase weediness through enhanced plant size. Selection acted simultaneously on multiple traits, with both linear and quadratic selection shaping phenotypic variation in both ruderal and agrestal environments, with no major differences between cross types. Selection promotes phenotypic diversity and might even promote genetic diversity and adaptation to new weed control strategies in agricultural habitats. Further research using advanced-generation hybrids is needed to confirm these findings. This is the first study providing a valuable contribution into how selection influences the evolutionary dynamics of weedy radish after intraspecific crop–weed hybridization.

## Supplementary Material

plag010_Supplementary_Data

## Data Availability

Dataset has been submitted to Figshare (2/11/2026). https://doi.org/10.6084/m9.figshare.31314307
