# Supplementary material for: Phenotypic selection in weedy radish and its bidirectional crop–weed hybrids across two contrasting environments
Source: AoB Plants. 2026 Feb 13;18(2):plag010. doi: 10.1093/aobpla/plag010 (PMC12957880; doi:10.1093/aobpla/plag010)
Supplement: plag010_Supplementary_Data [file plag010_supplementary_data.pdf]

TABLE S1. Correlations among plant traits and seed number in radish plants in Year 1.

	ELL	ELW	ELA	ERD	ELN	TTF	FLW	FLL	FLA	FLN	FPH	FSD	MPH	BN	LSD	RD	SN
Early leaf length (ELL)	1																
Early leaf width (ELW)	**0.92**	1															
Early leaf area (ELA)	**0.96**	**0.96**	1														
Rosette diameter (RSD)	**0.93**	**0.96**	**0.94**	1													
Rosette leaf number (RLN)	0.58	0.55	0.54	0.61	1												
Time to flowering (FT)	-0.31	-0.30	-0.28	-0.32	-0.34	1											
Leaf width at flowering (FLW)	0.78	0.73	0.74	0.75	0.47	-0.13	1										
Leaf length at flowering (FLL)	0.82	0.84	0.81	0.83	0.48	-0.08	0.86	1									
Leaf area at flowering (FLA)	0.83	0.81	0.83	0.81	0.47	-0.09	**0.93**	**0.95**	1								
Leaves number at flowering (FLN)	0.56	0.55	0.53	0.58	0.47	0.02	0.55	0.58	0.56	1							
Plant height at flowering (FPH)	0.69	0.75	0.70	0.72	0.38	0.13	0.65	0.81	0.74	0.60	1						
Stem diameter at flowering (FSD)	0.78	0.74	0.75	0.77	0.52	-0.17	0.84	0.81	0.83	0.65	0.65	1					
Plant height at maturity (MPH)	0.72	0.76	0.71	0.74	0.40	-0.04	0.75	0.85	0.79	0.56	0.84	0.75	1				
Branch numbers (BN)	0.63	0.63	0.60	0.65	0.46	-0.14	0.67	0.67	0.65	0.74	0.61	0.74	0.67	1			
Stem diameter at maturity (MSD)	0.74	0.70	0.71	0.73	0.49	-0.17	0.83	0.78	0.80	0.64	0.61	**0.93**	0.75	0.75	1		
Root diameter (RTD)	0.56	0.50	0.51	0.56	0.44	-0.01	0.66	0.60	0.63	0.64	0.47	0.75	0.58	0.66	0.79	1	
Seed number (SN)	0.51	0.48	0.48	0.50	0.34	-0.14	0.63	0.58	0.61	0.43	0.39	0.64	0.57	0.62	0.67	0.56	1

Correlation coefficients are reported. The pairwise correlations with r > |0.9| are in bold. N >500 for each combination

.TABLE S2. Correlations among plant traits and seed number in radish plants in Year 2.

	ELL	ELW	ELA	ERD	ELN	TTF	FLW	FLL	FLA	FLN	FPH	FSD	MPH	BN	LSD	RD	SN
Early leaf length (ELL)	1																
Early leaf width (ELW)	**0.90**	1															
Early leaf area (ELA)	**0.96**	**0.96**	1														
Rosette diameter (RSD)	**0.90**	**0.94**	**0.93**	1													
Rosette leaf number (RLN)	0.54	0.54	0.55	0.61	1												
Time to flowering (FT)	-0.24	-0.17	-0.19	-0.19	-0.23	1											
Leaf width at flowering (FLW)	0.71	0.72	0.71	0.73	0.47	-0.24	1										
Leaf length at flowering (FLL)	0.76	0.80	0.76	0.79	0.45	-0.21	**0.92**	1									
Leaf area at flowering (FLA)	0.72	0.75	0.75	0.75	0.45	-0.18	**0.95**	**0.95**	1								
Leaves number at flowering (FLN)	0.55	0.58	0.58	0.62	0.57	-0.18	0.68	0.63	0.64	1							
Plant height at flowering (FPH)	0.74	0.76	0.72	0.75	0.41	-0.18	0.80	**0.92**	0.81	0.52	1						
Stem diameter at flowering (FSD)	0.73	0.73	0.73	0.75	0.51	-0.26	0.92	**0.89**	**0.91**	0.73	0.81	1					
Plant height at maturity (MPH)	0.70	0.72	0.68	0.71	0.39	-0.16	0.80	**0.89**	0.79	0.53	**0.94**	0.79	1				
Branch numbers (BN)	0.50	0.51	0.53	0.56	0.50	-0.20	0.65	0.58	0.61	0.79	0.48	0.72	0.52	1			
Stem diameter at maturity (MSD)	0.68	0.70	0.70	0.72	0.50	-0.20	0.87	0.85	0.86	0.73	0.77	**0.92**	0.80	0.79	1		
Root diameter (RTD)	0.38	0.39	0.41	0.44	0.43	-0.08	0.53	0.43	0.48	0.72	0.31	0.59	0.35	0.74	0.67	1	
Seed number (SN)	0.42	0.00	0.45	0.47	0.38	-0.14	0.67	0.56	0.64	0.64	0.44	0.71	0.47	0.76	0.78	0.72	1

Correlation coefficients are reported. The pairwise correlations with r > |0.9| are in bold. N >500 for each combination

TABLE S3. Restricted maximum likelihood for ten traits: Rosette diameter (RSD), rosette leaf number (RLN), time to flowering (FT), leaf area at flowering (FLA), leaf number at flowering (FLN), plant height at flowering (FPH), stem diameter at flowering (FSD), plant height at maturity (MPH), branch number (BN), and root diameter (RTD), evaluated in bidirectional crop–weed hybrids and their progenitors (weeds and crop) of *Raphanus sativus* across two environments (ruderal and agrestal), using SAS PROC GLIMMIX in Year 1. Data were collected in the Agronomy Department at Universidad Nacional del Sur, Bahía Blanca, Argentina. dfn/dfd: numerator and denominator degrees of freedom. Significant differences in fixed effects are in bold.

**Year 1**		RSD		RLN		FT		FLA		FLN	
Fixed effects	df_n/df_d	*F*	*P*	*F*	*P*	*F*	*P*	*F*	*P*	*F*	*P*
Environment (E)	1/6	**109.72**	**<.0001**	**67.81**	**0.0002**	**12.70**	**0.0119**	**90.97**	**<.0001**	**99.38**	**<.0001**
Cross type (CT)	3/6	**12.45**	**0.0055**	**12.38**	**0.0056**	**48.22**	**0.0001**	**20.36**	**0.0015**	**26.97**	**0.0007**
E x C	3/6	0.5	0.6984	3.36	0.0962	3.65	0.0829	0.99	0.4601	**10.13**	**0.0092**
Random Effects		*Z*	*P*	*Z*	*P*	*Z*	*P*	*Z*	*P*	*Z*	*P*
Block (E)		2.16	0.0152	1.72	0.0425	2.03	0.0211	2.23	0.0128	1.22	0.1108
Biotype (CT)		1.22	0.112	.	.	0.91	0.1803	0.7	0.2428	.	.
Plants (E*Biotype*Block)		5.38	<.0001	1.5	0.0673	4.47	<.0001	16.62	<.0001	2.3	0.0108
Residual		.	.	.	.	.	.	.	.	.	.

		FPH		FST		MPH		BN		RTD	
Fixed effects	df_n/df_d	*F*	*P*	*F*	*P*	*F*	*P*	*F*	*P*	*F*	*P*
Environment (E)	1/6	**283.62**	**<.0001**	**83.18**	**<.0001**	**177.19**	**<.0001**	**69.94**	**0.0002**	**17.56**	**0.0057**
Cross type (CT)	3/6	**51.28**	**0.0001**	**30.39**	**0.0005**	**42.02**	**0.0002**	**36.36**	**0.0003**	**92.76**	**<.0001**
E x C	3/6	1.6	0.2847	4.16	0.0652	**7.75**	**0.0174**	4.72	0.0507	0.33	0.8031
Random Effects		*Z*	*P*	*Z*	*P*	*Z*	*P*	*Z*	*P*	*Z*	*P*
Block (E)		2.02	0.0215	1.93	0.0265	2.18	0.0148	1.77	0.0385	1.89	0.0291
Biotype (C*T)		0.19	0.426	.	.	0.18	0.4298	.	.	0.1	0.4621
Plants (E*Biotype*Block)		8.83	<.0001	2.01	0.022	8.78	<.0001	5.94	<.0001	0.26	0.3988
Residual		.	.	.	.	.	.	.	.	.	.

TABLE S4. Restricted maximum likelihood for ten traits: Rosette diameter (RSD), rosette leaf number (RLN), time to flowering (FT), leaf area at flowering (FLA), leaf number at flowering (FLN), plant height at flowering (FPH), stem diameter at flowering (FSD), plant height at maturity (MPH), branch number (BN), and root diameter (RTD), evaluated in bidirectional crop–weed hybrids and their progenitors (weeds and crop) of *Raphanus sativus* across two environments (ruderal and agrestal), using SAS PROC GLIMMIX in Year 2. Data were collected in the Agronomy Department at Universidad Nacional del Sur, Bahía Blanca, Argentina. dfn/dfd: numerator and denominator degrees of freedom. Significant differences in fixed effects are in bold.

**Year 2**		RSD		RLN		FT		FLA		FLN	
Fixed effects	df_n/df_d	*F*	*P*	*F*	*P*	*F*	*P*	*F*	*P*	*F*	*P*
Environment (E)	**1/6**	**208.31**	**<.0001**	**11.22**	**0.0154**	2.61	0.1575	**216.48**	**<.0001**	**16.26**	**0.0069**
Cross type (CT)	**3/6**	**26.08**	**0.0008**	**9.07**	**0.012**	0.50	0.6939	**40.8**	**0.0002**	**126.81**	**<.0001**
E x C	**3/6**	4.62	0.053	2.62	0.1457	0.15	0.9253	**10.66**	**0.0081**	1.33	0.3491
Random Effects		*Z*	*P*	*Z*	*P*	*Z*	*P*	*Z*	*P*	*Z*	*P*
Block (E)		1.37	0.0854	2.19	0.0144	2.33	0.0098	1.92	0.0273	2.16	0.0154
Biotype (CT)		0.41	0.3403	0.86	0.195	1.54	0.0613	.	.	.	.
Plants (E*Biotype*Block)		1.78	0.0374	.	.	.	.	0.23	0.4093	0.2	0.4211
Residual		1.02	0.1547	17.38	<.0001	22.67	<.0001	1.35	0.0886	0.38	0.3525

		FPH		FST		MPH		BN		RTD	
Fixed effects	df_n/df_d	*F*	*P*	*F*	*P*	*F*	*P*	*F*	*P*	*F*	*P*
Environment (E)	**1/6**	**486.54**	**<.0001**	**94.15**	**<.0001**	**494.68**	**<.0001**	**7.56**	**0.0333**	0.06	0.8127
Cross type (CT)	**3/6**	**12.52**	**0.0054**	**44.83**	**0.0002**	**38.61**	**0.0003**	**77.88**	**<.0001**	**41.63**	**0.0002**
E x C	**3/6**	2.02	0.2125	**5.71**	**0.0343**	3.29	0.0997	2.92	0.1223	1.25	0.3711
Random Effects		*Z*	*P*	*Z*	*P*	*Z*	*P*	*Z*	*P*	*Z*	*P*
Block (E)		1.65	0.049	2.15	0.0159	1.93	0.027	2.25	0.0123	2.29	0.0109
Biotype (CT)		1.22	0.1114	0.24	0.4049	0.74	0.2297	0.07	0.4726	1.08	0.1401
Plants (E*Biotype*Block)		.	.	5.32	<.0001	.	.	5.64	<.0001	0.61	0.2708
Residual		17.38	<.0001	0.67	0.2522	17.24	<.0001	0.67	0.2515	0.54	0.2943

TABLE S5. Levene's tests for homogeneity/differences in phenotypic variance among cross types: bidirectional crop (C)–weed (W) hybrids (CxW and WxC) and their progenitors (weeds and crop) of *Raphanus sativus* across two environments (ruderal and agrestal), using SAS PROC GLM in Year 1 and 2, for ten traits: Rosette diameter (RSD), rosette leaf number (RLN), time to flowering (FT), leaf area at flowering (FLA), leaf number at flowering (FLN), plant height at flowering (FPH), stem diameter at flowering (FSD), plant height at maturity (MPH), branch number (BN), and root diameter (RTD). Data were collected in the Agronomy Department at Universidad Nacional del Sur, Bahía Blanca, Argentina. Significant differences in fixed effects are in bold.

	Year 1				Year 2			
	Ruderal		Agrestal		Ruderal		Agrestal	
Traits	*F*	*P*	*F*	*P*	*F*	*P*	*F*	*P*
RSD	**3.10**	**0.0271**	0.16	0.9212	1.66	0.1745	**5.29**	**0.0014**
RLN	0.47	0.7036	1.34	0.2626	1.64	0.1794	**3.34**	**0.0196**
FT	1.94	0.1215	0.63	0.5979	2.40	0.0668	**3.22**	**0.0224**
FLA	**4.70**	**0.0032**	1.85	0.1376	1.59	0.1911	**5.67**	**0.0009**
FLN	**3.97**	**0.0085**	**2.78**	**0.0411**	2.17	0.0911	**7.78**	**<0.0001**
FPH	**4.32**	**0.0053**	**5.61**	**0.0009**	1.60	0.1895	**4.54**	**0.0039**
FSD	**6.10**	**0.0005**	2.32	0.0755	0.50	0.6851	**4.65**	**0.0034**
MPH	**3.37**	**0.0189**	0.37	0.7719	0.46	0.7088	1.93	0.1248
BN	1.60	0.1890	**5.71**	**0.0008**	2.02	0.1116	1.93	0.1249
RTD	**13.43**	**<0.0001**	**8.77**	**<0.0001**	**4.00**	**0.0082**	**9.98**	**<0.0001**

TABLE S6. Magnitude of phenotypic variation among cross types expressed as coefficients of variation (CV, %) of functional traits for ten traits: Rosette diameter (RSD), rosette leaf number (RLN), time to flowering (FT), leaf area at flowering (FLA), leaf number at flowering (FLN), plant height at flowering (FPH), stem diameter at flowering (FSD), plant height at maturity (MPH), branch number (BN), and root diameter (RTD), evaluated in bidirectional crop–weed hybrids and their progenitors (weeds and crop) of *Raphanus sativus* across two environments (ruderal and agrestal), in Year 1 and 2. CV were calculated for each trait within each environment, year, and cross type as the ratio of the standard deviation to the mean, multiplied by 100. Data were collected in the Agronomy Department at Universidad Nacional del Sur, Bahía Blanca, Argentina. Cross types with significant Levene's tests are in bold.

	Year 1							
	Ruderal				Agrestal			
Traits	C	CxW	WxC	W	C	CxW	WxC	W
RSD	**27.2**	**28.1**	**31.3**	**31.4**	29.3	26.3	22.9	26.1
RLN	23.9	18.4	18.1	18.4	13.5	15.1	15.0	16.3
FT	10.5	9.9	8.7	10.1	7.7	8.2	7.2	7.4
FLA	**95.2**	**62.3**	**70.2**	**79.8**	51.0	44.1	45.1	43.7
FLN	**32.3**	**20.9**	**18.2**	**22.2**	**19.1**	**19.1**	**18.2**	**16.4**
FPH	**36.5**	**30.4**	**29.6**	**33.0**	**23.7**	**17.5**	**20.1**	**25.7**
FSD	**38.9**	**38.5**	**42.9**	**41.4**	27.4	30.8	24.0	25.7
MPH	**22.1**	**22.5**	**26.3**	**27.3**	12.8	11.5	13.4	14.0
BN	87.6	44.0	46.0	54.8	**47.9**	**28.4**	**29.5**	**26.1**
RTD	**51.8**	**51.6**	**53.7**	**45.3**	**30.8**	**39.5**	**36.4**	**29.9**

	Year 2							
	Ruderal				Agrestal			
Traits	C	CxW	WxC	W	C	CxW	WxC	W
RSD	21.8	18.1	17.2	19.2	**26.0**	**28.2**	**29.7**	**27.6**
RLN	20.8	19.4	15.2	16.6	**21.3**	**18.3**	**18.3**	**17.2**
FT	7.2	7.3	7.5	10.1	**4.8**	**5.6**	**5.8**	**7.0**
FLA	46.1	54.9	56.8	69.4	**68.7**	**61.4**	**59.1**	**66.7**
FLN	24.4	20.0	17.8	27.6	**27.3**	**25.8**	**21.8**	**22.7**
FPH	25.5	21.8	32.2	37.0	**16.8**	**15.1**	**22.5**	**20.2**
FSD	27.0	27.7	27.4	32.2	**36.9**	**32.8**	**32.7**	**28.8**
MPH	19.0	18.6	21.7	27.9	18.5	12.0	18.1	16.8
BN	51.4	34.6	36.3	48.5	42.6	35.0	37.3	52.0
RTD	**47.1**	**45.2**	**45.1**	**51.9**	**63.4**	**44.8**	**45.7**	**37.6**

TABLE S7. Restricted maximum likelihood for direct directional selection (β') of ten traits: Rosette diameter (RSD), rosette leaf number (RLN), time to flowering (FT), leaf area at flowering (FLA), leaf number at flowering (FLN), plant height at flowering (FPH), stem diameter at flowering (FSD), plant height at maturity (MPH), branch number (BN), and root diameter (RTD), evaluated in three cross types (weed and bidirectional crop–weed hybrids) of *Raphanus sativus* across two environments (ruderal and agrestal), using SAS PROC GLIMMIX in Year 1. Data were collected in the Agronomy Department at Universidad Nacional del Sur, Bahía Blanca, Argentina. dfn/dfd: numerator and denominator degrees of freedom. Significant differences in fixed effects are in bold.

Year 1 - β'			
Fixed effects	df_n/df_d	*F*	*P*
RSD	1/457	0.51	0.4776
RSD*Environment (E)	1/457	**7.15**	**0.0078**
RSD*Cross type (CT)	2/457	1.83	0.1618
RSD*E*CT	2/457	1.73	0.1779
RLN	1/457	0.42	0.5174
RLN*E	1/457	**12.59**	**0.0004**
RLN*CT	2/457	0.19	0.8255
RLN*E*CT	2/457	0.16	0.8524
FT	1/457	0.04	0.8391
FT*E	1/457	0.79	0.3735
FT*CT	2/457	1.09	0.3359
FT*E*CT	2/457	0.31	0.7365
FLA	1/457	**20.74**	**<0.0001**
FLA*E	1/457	**5.31**	**0.0216**
FLA*CT	2/457	0.01	0.9936
FLA*E*CT	2/457	0.23	0.7972
FLN	1/457	1.61	0.2048
FLN*E	1/457	2.64	0.1047
FLN*CT	2/457	0.31	0.7351
FLN*E*CT	2/457	0.55	0.5746
FPH	1/457	**26.55**	**<0.0001**
FPH*E	1/457	0.32	0.5701
FPH*CT	2/457	1.32	0.2671
FPH*E*CT	2/457	1.96	0.1422
FSD	1/457	1.87	0.1722
FSD*E	1/457	2.24	0.1355
FSD*CT	2/457	2.34	0.0975
FSD*E*CT	2/457	0.13	0.8798
MPH	1/457	**14.56**	**0.0002**
MPH*E	1/457	0.90	0.3442
MPH*CT	2/457	1.61	0.2012
MPH*E*CT	2/457	2.03	0.1327
BN	1/457	**17.66**	**<0.0001**
BN*E	1/457	0.65	0.4204
BN*CT	2/457	0.01	0.9858
BN*E*CT	2/457	0.25	0.7784
RTD	1/457	0.83	0.3616
RTD*E	1/457	3.63	0.0574
RTD*CT	2/457	**3.93**	**0.0204**
RTD*E*CT	2/457	0.92	0.3999
Random Effects		*Z*	*P*
Block (E)		1.54	0.0613
Biotipo (CT)		0.75	0.2254
Residual		15.08	<.0001

TABLE S8. Restricted maximum likelihood for direct directional selection (β') of ten traits: Rosette diameter (RSD), rosette leaf number (RLN), time to flowering (FT), leaf area at flowering (FLA), leaf number at flowering (FLN), plant height at flowering (FPH), stem diameter at flowering (FSD), plant height at maturity (MPH), branch number (BN), and root diameter (RTD), evaluated in three cross types (weed and bidirectional crop–weed hybrids) of *Raphanus sativus* across two environments (ruderal and agrestal), using SAS PROC GLIMMIX in Year 2. Data were collected in the Agronomy Department at Universidad Nacional del Sur, Bahía Blanca, Argentina. dfn/dfd: numerator and denominator degrees of freedom. Significant differences in fixed effects are in bol

Year 2 - β'			
Fixed effects	df_n/df_d	*F*	*P*
RSD	1/436	**5.28**	**0.0221**
RSD*Environment (E)	1/436	1.55	0.2136
RSD*Cross type (CT)	2/436	0.78	0.4569
RSD*E*CT	2/436	1.37	0.2559
RLN	1/436	0.26	0.6081
RLN*E	1/436	0.99	0.3199
RLN*CT	2/436	0.43	0.6536
RLN*E*CT	2/436	0.54	0.5830
FT	1/436	0.52	0.4706
FT*E	1/436	**13.83**	**0.0002**
FT*CT	2/436	0.72	0.4854
FT*E*CT	2/436	1.24	0.2893
FLA	1/436	**15.19**	**0.0001**
FLA*E	1/436	3.55	0.0602
FLA*CT	2/436	0.21	0.8111
FLA*E*CT	2/436	0.73	0.4838
FLN	1/436	**9.40**	**0.0023**
FLN*E	1/436	0.26	0.6102
FLN*CT	2/436	0.53	0.5907
FLN*E*CT	2/436	0.54	0.5831
FPH	1/436	**18.08**	**<0.0001**
FPH*E	1/436	0.00	0.9761
FPH*CT	2/436	2.97	0.0523
FPH*E*CT	2/436	0.19	0.8245
FSD	1/436	**14.76**	**0.0001**
FSD*E	1/436	0.00	0.9768
FSD*CT	2/436	0.77	0.4627
FSD*E*CT	2/436	0.58	0.5626
MPH	1/436	0.00	0.9830
MPH*E	1/436	0.03	0.8580
MPH*CT	2/436	1.04	0.3544
MPH*E*CT	2/436	2.34	0.0980
BN	1/436	**50.05**	**<0.0001**
BN*E	1/436	**4.74**	**0.0299**
BN*CT	2/436	**3.34**	**0.0363**
BN*E*CT	2/436	0.25	0.7814
RTD	1/436	**40.03**	**<0.0001**
RTD*E	1/436	1.77	0.1838
RTD*CT	2/436	0.67	0.5138
RTD*E*CT	2/436	2.55	0.0794
Random Effects		*Z*	*P*
Block (E)		.	.
Biotipo (CT)		0.44	0.3285
Residual		15.06	<0.0001

TABLE S9. Restricted maximum likelihood for direct nonlinear selection (γ') of ten traits: Rosette diameter (RSD), rosette leaf number (RLN), time to flowering (FT), leaf area at flowering (FLA), leaf number at flowering (FLN), plant height at flowering (FPH), stem diameter at flowering (FSD), plant height at maturity (MPH), branch number (BN), and root diameter (RTD), evaluated in three cross types (weed and bidirectional crop–weed hybrids) of *Raphanus sativus* across two environments (ruderal and agrestal), using SAS PROC GLIMMIX in Year 1. Data were collected in the Agronomy Department at Universidad Nacional del Sur, Bahía Blanca, Argentina. dfn/dfd: numerator and denominator degrees of freedom. Significant differences in fixed effects are in bold.

Year 1 - γ'_{ii}			
Fixed effects	df_n/df_d	*F*	*P*
RSD	1/457	0.79	0.3741
RSD*Environment (E)	1/457	3.08	0.0798
RSD*Cross type (CT)	2/457	**4.23**	**0.0152**
RSD*E*CT	2/457	**5.80**	**0.0033**
RLN	1/457	**7.26**	**0.0073**
RLN*E	1/457	**9.00**	**0.0029**
RLN*CT	2/457	0.30	0.7406
RLN*E*CT	2/457	0.25	0.7820
FT	1/457	0.35	0.5518
FT*E	1/457	1.60	0.2064
FT*CT	2/457	0.11	0.8968
FT*E*CT	2/457	1.22	0.2970
FLA	1/457	**8.47**	**0.0038**
FLA*E	1/457	**12.61**	**0.0004**
FLA*CT	2/457	1.68	0.1872
FLA*E*CT	2/457	1.44	0.2372
FLN	1/457	**9.90**	**0.0018**
FLN*E	1/457	**3.96**	**0.0473**
FLN*CT	2/457	2.13	0.1200
FLN*E*CT	2/457	**4.18**	**0.0158**
FPH	1/457	**8.82**	**0.0031**
FPH*E	1/457	1.39	0.2394
FPH*CT	2/457	0.73	0.4830
FPH*E*CT	2/457	0.93	0.3966
FSD	1/457	**43.66**	**<0.0001**
FSD*E	1/457	**33.48**	**<0.0001**
FSD*CT	2/457	0.93	0.3946
FSD*E*CT	2/457	1.12	0.3261
MPH	1/457	0.72	0.3979
MPH*E	1/457	**5.67**	**0.0177**
MPH*CT	2/457	**3.24**	**0.0402**
MPH*E*CT	2/457	1.65	0.1931
BN	1/457	3.86	0.0500
BN*E	1/457	3.84	0.0506
BN*CT	2/457	0.41	0.6614
BN*E*CT	2/457	0.08	0.9270
RTD	1/457	0.44	0.5066
RTD*E	1/457	2.14	0.1439
RTD*CT	2/457	0.53	0.5874
RTD*E*CT	2/457	0.41	0.6670
Random Effects		*Z*	*P*
Block (E)		1.64	0.0507
Biotipo (CT)		0.19	0.4259
Residual		15.15	<0.0001

TABLE S10. Restricted maximum likelihood for direct nonlinear selection (γ') of ten traits: Rosette diameter (RSD), rosette leaf number (RLN), time to flowering (FT), leaf area at flowering (FLA), leaf number at flowering (FLN), plant height at flowering (FPH), stem diameter at flowering (FSD), plant height at maturity (MPH), branch number (BN), and root diameter (RTD), evaluated in three cross types (weed and bidirectional crop–weed hybrids) of *Raphanus sativus* across two environments (ruderal and agrestal), using SAS PROC GLIMMIX in Year 2. Data were collected in the Agronomy Department at Universidad Nacional del Sur, Bahía Blanca, Argentina. dfn/dfd: numerator and denominator degrees of freedom. Significant differences in fixed effects are in bold.

Year 2 - γ'_{ii}			
Fixed effects	df_n/df_d	*F*	*P*
RSD	1/436	2.02	0.1555
RSD*Environment (E)	1/436	**7.01**	**0.0084**
RSD*Cross type (CT)	2/436	0.24	0.7890
RSD*E*CT	2/436	1.56	0.2113
RLN	1/436	0.04	0.8377
RLN*E	1/436	0.42	0.5159
RLN*CT	2/436	0.14	0.8720
RLN*E*CT	2/436	1.49	0.2270
FT	1/436	**8.28**	**0.0042**
FT*E	1/436	**6.87**	**0.0091**
FT*CT	2/436	**3.79**	**0.0233**
FT*E*CT	2/436	2.38	0.0935
FLA	1/436	**10.06**	**0.0016**
FLA*E	1/436	**47.45**	**<0.0001**
FLA*CT	2/436	**3.43**	**0.0331**
FLA*E*CT	2/436	**3.79**	**0.0233**
FLN	1/436	3.28	0.0707
FLN*E	1/436	0.50	0.4813
FLN*CT	2/436	0.56	0.5706
FLN*E*CT	2/436	0.34	0.7104
FPH	1/436	0.61	0.4366
FPH*E	1/436	2.87	0.0907
FPH*CT	2/436	0.37	0.6932
FPH*E*CT	2/436	0.83	0.4389
FSD	1/436	0.79	0.3742
FSD*E	1/436	4.35	0.0377
FSD*CT	2/436	0.50	0.6064
FSD*E*CT	2/436	1.92	0.1483
MPH	1/436	**7.71**	**0.0057**
MPH*E	1/436	**6.09**	**0.0140**
MPH*CT	2/436	1.48	0.2283
MPH*E*CT	2/436	2.35	0.0966
BN	1/436	0.11	0.7445
BN*E	1/436	0.77	0.3805
BN*CT	2/436	1.63	0.1970
BN*E*CT	2/436	0.26	0.7674
RTD	1/436	0.04	0.8365
RTD*E	1/436	**5.29**	**0.0219**
RTD*CT	2/436	2.22	0.1102
RTD*E*CT	2/436	0.82	0.4414
Random Effects		*Z*	*P*
Block (E)		1.09	0.1387
Biotipo (CT)		1.62	0.0529
Residual		14.71	<0.0001

TABLE S11. Quadratic selection coefficients for ten traits: Rosette diameter (RSD), rosette leaf number (RLN), time to flowering (FT), leaf area at flowering (FLA), leaf number at flowering (FLN), plant height at flowering (FPH), stem diameter at flowering (FSD), plant height at maturity (MPH), branch number (BN), and root diameter (RTD), for the interaction between three cross types (CT): weed (W) and bidirectional crop–weed hybrids (W x C and C x W) of *Raphanus sativus* and two environments (E): ruderal (R) and agrestal (A), using restricted maximum likelihood in SAS PROC GLIMMIX in Year 1.

	By E x CT																		
		R - W			R - W x C			R - C x W			A - W			A - W x C			A - C x W		
Traits	Sig int	$\gamma'_{ii} \pm SE$			$\gamma'_{ii} \pm SE$			$\gamma'_{ii} \pm SE$			$\gamma'_{ii} \pm SE$			$\gamma'_{ii} \pm SE$			$\gamma'_{ii} \pm SE$		
RSD	**	0.190	0.080	*	0.048	0.132	ns	0.154	0.182	ns	0.030	0.087	ns	0.085	0.107	ns	-0.233	0.115	*
RLN	ns	-	-	-	-	-	-	-	-	-	-	-	-	-	-	-	-	-	-
FT	ns	-	-	-	-	-	-	-	-	-	-	-	-	-	-	-	-	-	-
FLA	ns	-	-	-	-	-	-	-	-	-	-	-	-	-	-	-	-	-	-
FLN	*	-0.086	0.057	ns	-0.149	0.155	ns	0.032	0.099	ns	-0.226	0.139	ns	0.016	0.101	ns	0.061	0.122	ns
FPH	ns	-	-	-	-	-	-	-	-	-	-	-	-	-	-	-	-	-	-
FSD	ns	-	-	-	-	-	-	-	-	-	-	-	-	-	-	-	-	-	-
MPH	ns	-	-	-	-	-	-	-	-	-	-	-	-	-	-	-	-	-	-
BN	ns	-	-	-	-	-	-	-	-	-	-	-	-	-	-	-	-	-	-
RTD	ns	-	-	-	-	-	-	-	-	-	-	-	-	-	-	-	-	-	-

TABLE S12. Quadratic selection coefficients for ten traits: Rosette diameter (RSD), rosette leaf number (RLN), time to flowering (FT), leaf area at flowering (FLA), leaf number at flowering (FLN), plant height at flowering (FPH), stem diameter at flowering (FSD), plant height at maturity (MPH), branch number (BN), and root diameter (RTD), for the interaction between three cross types (CT): weed (W) and bidirectional crop–weed hybrids (W x C and C x W) of *Raphanus sativus* and two environments (E): ruderal (R) and agrestal (A), using restricted maximum likelihood in SAS PROC GLIMMIX in Year 2.

	By E x CT																		
		R - W			R - W x C			R - C x W			A - W			A - W x C			A - C x W		
Traits	Sig int	$\gamma'_{ii} \pm$ SE			$\gamma'_{ii} \pm$ SE			$\gamma'_{ii} \pm$ SE			$\gamma'_{ii} \pm$ SE			$\gamma'_{ii} \pm$ SE			$\gamma'_{ii} \pm$ SE		
RSD	ns	-	-	-	-	-	-	-	-	-	-	-	-	-	-	-	-	-	-
RLN	ns	-	-	-	-	-	-	-	-	-	-	-	-	-	-	-	-	-	-
FT	ns	-	-	-	-	-	-	-	-	-	-	-	-	-	-	-	-	-	-
FLA	*	0.474	0.462	ns	-1.808	1.002	ns	-0.395	0.745	ns	0.026	0.080	ns	0.228	0.113	*	0.384	0.114	**
FLN	ns	-	-	-	-	-	-	-	-	-	-	-	-	-	-	-	-	-	-
FPH	ns	-	-	-	-	-	-	-	-	-	-	-	-	-	-	-	-	-	-
FSD	ns	-	-	-	-	-	-	-	-	-	-	-	-	-	-	-	-	-	-
MPH	ns	-	-	-	-	-	-	-	-	-	-	-	-	-	-	-	-	-	-
BN	ns	-	-	-	-	-	-	-	-	-	-	-	-	-	-	-	-	-	-
RTD	ns	-	-	-	-	-	-	-	-	-	-	-	-	-	-	-	-	-	-
